# Supplementary material for: Microscope-Based Augmented Reality with Intraoperative Computed Tomography-Based Navigation for Resection of Skull Base Meningiomas in Consecutive Series of 39 Patients
Source: Cancers (Basel). 2022 May 6;14(9):2302. doi: 10.3390/cancers14092302 (PMC9101634; doi:10.3390/cancers14092302)
Supplement: Supplementary file 1 [file cancers-14-02302-s001.zip › cancers-1685965-supplementary.pdf]

Supplementary Materials: Microscope-based Augmented Reality with Intraoperative Computed Tomography-based Navigation for Resection of Skull base Meningiomas in Consecutive Series of 39 patients

Mirza Pojskić, Miriam Bopp, Benjamin Saβ, Barbara Carl and Christopher Nimsky

Four operative videos/Supplemental material (Google Drive link), with following description:

i. Patient number 9: Microsurgical resection of medial sphenoid wing meningioma using microscope-based augmented reality and intraoperative computed tomography-based navigation
ii. Pt 28:Microsurgical resection of right clinoidal meningioma via fronto-temporal craniotomy with microscope-based augmented reality
iii. Pt 31:Microsurgical resection of recurrent sphenoid wing meningioma using microscope-based augmented reality with intraoperative computed tomography
iv. Pt 36: Microsurgical resection of giant olfactory meningioma via bifrontal approach with use of augmented reality and intraoperative CT-based navigation

These videos can be found also on Zenodo, on the following link:
https://zenodo.org/record/6403303#.YkcfBzVCSUl
